# Terrestrial Morphotypes of Aquatic Plants Display Improved Seed Germination to Deal with Dry or Low-Rainfall Periods

**DOI:** 10.3390/plants10040741

**Published:** 2021-04-10

**Authors:** Rocío Fernández-Zamudio, Pablo García-Murillo, Carmen Díaz-Paniagua

**Affiliations:** 1 Doñana Biological Station-CSIC, 41092 Sevilla, Spain; rzamudio@ebd.csic.es; 2 Department of Plant Biology and Ecology, University of Sevilla, 41004 Sevilla, Spain; pgarcia@us.es

**Keywords:** seeds, germination, reproductive strategies, *Callitriche brutia*, *Ranunculus peltatus* subsp. *saniculifolius*, temporary ponds, Mediterranean wetlands

## Abstract

In temporary ponds, seed germination largely determines how well aquatic plant assemblages recover after dry periods. Some aquatic plants have terrestrial morphotypes that can produce seeds even in dry years. Here, we performed an experiment to compare germination patterns for seeds produced by aquatic and terrestrial morphotypes of *Ranunculus peltatus* subsp. *saniculifolius* over the course of five inundation events. During the first inundation event, percent germination was higher for terrestrial morphotype seeds (36.1%) than for aquatic morphotype seeds (6.1%). Seed germination peaked for both groups during the second inundation event (terrestrial morphotype: 47%; aquatic morphotype: 34%). Even after all five events, some viable seeds had not yet germinated (terrestrial morphotype: 0.6%; aquatic morphotype: 5%). We also compared germination patterns for the two morphotypes in *Callitriche brutia*: the percent germination was higher for terrestrial morphotype seeds (79.5%) than for aquatic morphotype seeds (41.9%). Both aquatic plant species use two complementary strategies to ensure population persistence despite the unpredictable conditions of temporary ponds. First, plants can produce seeds with different dormancy periods that germinate during different inundation periods. Second, plants can produce terrestrial morphotypes, which generate more seeds during dry periods, allowing for re-establishment when conditions are once again favorable.

## 1. Introduction

Aquatic plants form a species-rich group whose members display marked differences in morphology, physiology, reproduction, and life history. Another source of variation is inundation tolerance. Aquatic plants include, but are not limited to, terrestrial species able to persist in flooded soils, amphibious species that can grow equally well on land or in water, and aquatic species that only grow in water [1]. Most aquatic plants can use asexual and sexual reproduction. In permanent water bodies, where habitat conditions remain constant, asexual reproduction is favored, while seed production may be rare or absent [2]. In contrast, sexual reproduction appears to be more advantageous in changing or fluctuating environments [3]. Temporary ponds cycle through wet and dry phases, leading to unstable habitat conditions. Consequently, aquatic organisms living in temporary ponds have developed strategies for coping with dry periods. In aquatic plants, seed germination allows plant populations to regenerate during subsequent wet phases. In Mediterranean ecosystems, the timing and quantity of precipitation can be highly unpredictable. It is common for years of heavy rains, during which ponds display very long hydroperiods (>8 months), to alternate with very dry years, during which ponds are ephemeral or may not even flood [4,5]. In temporary wetlands found in regions with warm, dry summers, aquatic plants cannot re-establish themselves via asexual propagules and mainly depend on seed production and the formation of a persistent seed bank [6,7,8,9,10,11,12,13]. In Mediterranean temporary ponds, unpredictable rainfall can be catastrophic for submerged plants if flooding triggers seed germination, but the hydroperiod does not last long enough for plants to produce a new generation of seeds. After such “bad years”, seed banks can help re-establish aquatic plant populations in future “good years” [14,15], thus reducing the risk of plant extinction [16].

In response to these unpredictable inundation regimes, some plant species display marked plasticity. They can switch from aquatic to terrestrial morphotypes following drastic drops in water level [17,18]. They can also develop terrestrial morphotypes capable of withstanding dry periods and producing flowers, fruits, and seeds [18,19]. Many aquatic species, especially those in temporary wetlands, develop terrestrial morphotypes [20,21,22,23,24]. Brock and Casanova [18] referred to such species as “amphibious”. This group includes “fluctuation responders”—species with pronounced plasticity that may change morphotypes to persist in the absence of water. Deil [9] described “dwarf annuals with ephemeroid syndrome,” which are plants that adopt morphotypes adapted to temporary flooding. More specifically, they are species with a high degree of phenotypic plasticity that display a tendency toward nanism and an ability to produce fruit just a few weeks after germination. Plants from the families Callitrichaceae and Ranunculaceae are included in this group.

In our study, we aimed to analyze the reproductive strategies that allow submerged plants to cope with adverse periods, during which inundation is rare or absent. We conducted an experiment to assess the germination dynamics of two species commonly found in Mediterranean temporary ponds: *Ranunculus peltatus* subsp. *saniculifolius* and *Callitriche brutia*. Both species have aquatic and terrestrial morphotypes, and we compared the germination patterns of seeds produced by the two groups. Because these species frequently occur in ponds with recurrent dry periods, we hypothesized that seeds could play an important role in plant re-establishment following dry phases. We thus expected to observe high levels of seed germination and/or viability. Our main objective was to assess how the seed germination patterns of aquatic and terrestrial morphotypes could contribute to the long-term persistence of these two species. More specifically, we aimed to (1) compare the percent germination of seeds produced by aquatic and terrestrial morphotypes, (2) explore the response of seed banks to inundation/desiccation cycles, and (3) evaluate the viability of ungerminated seeds within the seed bank and the potential impact on the long-term persistence of the seed bank.

## 2. Methods

### 2.1. Study Area

**Figure 1:**
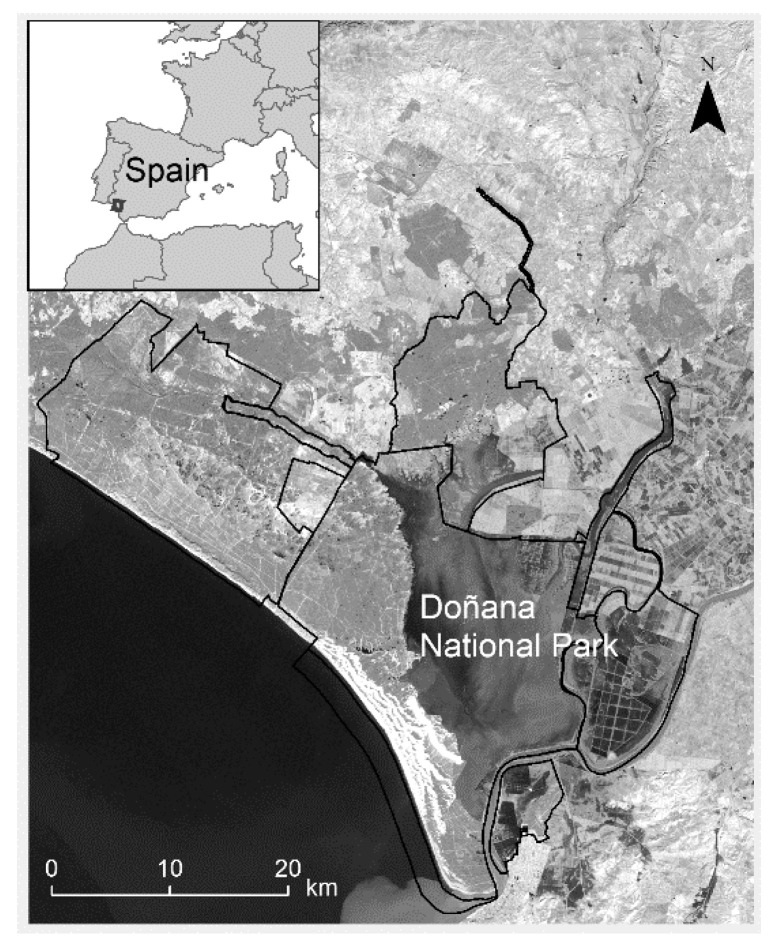
Location of the study area.

Our study was carried out on submerged plant species that commonly occur in the temporary ponds of Doñana National Park (Figure 1), a protected area located in southwestern Spain (36°59′19″ N/6°26′35″ W). The climate in this region is Mediterranean, with mild winters, hot summers (mean daily temperature for 1978–2020 data: annual = 17.1 °C, of the coldest month (January) = 10 °C, of the warmest month (August) = 24.5 °C), and a highly uneven distribution of rain from autumn to spring (mean annual rainfall: 550 L). In very rainy years, it is possible for more than 3000 temporary ponds to form in Doñana. Pond inundation cycles differ widely across years depending on the seasonal concentration of rain. In some years, ponds fill in the autumn or winter and have hydroperiods of 6–9 months. In other years, which may be drier, ponds flood in the spring and have hydroperiods of 3–5 months. Most ponds dry up in the summer [4,5]. This marked heterogeneity in pond hydroperiod favors high plant diversity (i.e., >200 species; [25,26]). 

### 2.2. Study Species

**Figure 2:**
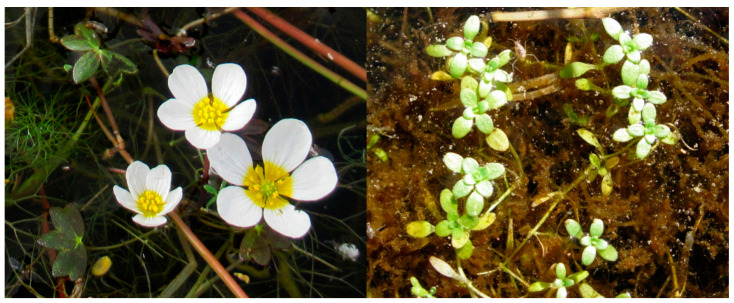
The studied species: *Ranunculus peltatus* subsp. *saniculifolius* (**left**) and *Callitriche brutia* (**right**).

Our two study species are part of taxonomic groups whose delimitation is controversial: *Ranunculus* subgen. *Batrachium* and *Callitriche* [27,28,29,30,31]. In this study, we used the classification scheme in Cirujano et al. [32]: the *Ranunculus* species corresponded to *Ranunculus peltatus* subsp. *saniculifolius* (Viv.) CDK Cook, and the *Callitriche* species corresponded to *Callitriche brutia* Petagna (Figure 2). They are typical aquatic species in Mediterranean temporary wetlands and the most common type of dormancy for these types of species is physical dormancy [33].

We analyzed the seed germination patterns of these two species, which commonly form a dense submerged layer of vegetation that is characteristic of meso-oligotrophic (i.e., nutrient-poor) ponds in the Doñana pond network [26].

In the case of *Ranunculus peltatus* subsp. *saniculifolius*, we studied seed germination across five consecutive inundation events. This species can be an annual or a perennial and usually exists in its aquatic morphotype, submerged in seasonal or permanent sources of still or running water [32,34]. Like other members of the genus *Ranunculus*, this species displays pronounced morphological plasticity and may shift from an aquatic to a terrestrial morphotype in response to changes in water level, environmental conditions, and hydrological regimes, among other factors [17,27,35]. In its aquatic morphotype, the plant has long, erect stems with capillary leaves that spread beneath the water surface. On the water surface, the plant forms laminary leaves and pedunculated flowers; it eventually produces globose fruits with 10–25 achenes. In its terrestrial morphotype, the plant is caespitose with prostrate tails measuring around 4 cm. Patterns of flower and fruit production are generally similar in both forms; however, flowers are produced over a longer period in the terrestrial versus the aquatic morphotype.

The second study species, *Callitriche brutia*, is a delicate plant that is commonly found in still, shallow waters [32]. It most frequently has long, thin stems bearing long, narrow leaves that are found underneath the water surface and a small rosette of 7–10 leaves that floats on the water surface [30]. Its small, elliptical fruits (<1.2 mm in length) occur in the axils of the submerged leaves. In contrast, the terrestrial morphotype has pedunculate fruits, which facilitate the seeds’ penetration into the soil [30].

### 2.3. Experimental Work

To analyze germination patterns in *R. peltatus* subsp. *sanicunifolius*, we collected seeds from naturally occurring plants in the spring of 2012, a low-rainfall year during which very few temporary ponds in the study area flooded. We sampled individuals with the aquatic morphotype in inundated temporary ponds and individuals with the terrestrial morphotype in dry temporary ponds. We took a single fruit from 29 plants in each group; the total seed number differed between groups (aquatic morphotype: *n* = 328 seeds, mean number of seeds per fruit = 11; terrestrial morphotype: *n* = 423 seeds, mean number of seeds per fruit = 13.5). After collection, seeds were stored under dry conditions during the summer (mean temperature = 29.1 °C; temperature range = 25–33 °C). We then exposed the seeds to five inundation events (G1–G5). During each event, we placed 1–5 seeds in the wells of microtiter plates (24 wells/tray) on which the fruit of origin and plant morphotype were identified. The first inundation event (G1) began on 4 December 2012, when we filled the wells with dechlorinated tap water and put the plates in a climatic chamber (light cycle: 12 h/12 h; temperature: 20 °C). We then checked the plates daily until we observed the first germinated seed. The plates were subsequently verified twice a week until three weeks had passed without any further signs of germination. On each monitoring day, we identified, counted, and extracted the seedlings, recording the day of germination. We retained all the ungerminated seeds for subsequent use. Between inundation events, there was a period of dry storage conducted under differing conditions (Table 1). After G1, the ungerminated seeds were stored for one month at 4 °C. The second inundation event (G2) began on 16 May 2013. The third inundation event (G3) started on 8 January 2014, after a dry storage period of the ungerminated seeds at room temperature (20–26 °C). The fourth inundation event (G4) began on 11 November 2014, after a dry storage period of the ungerminated seeds at 4 °C for one month. The fifth inundation event (G5) began on 8 May 2015, followed a dry storage period of the remaining seeds at 10 °C for one month. After the fifth inundation event, we dried and stored the remaining ungerminated seeds, and some months later, we assessed the viability of the seeds using tetrazolium (2,3,5-triphenyl-tetrazolium chloride) tests [36]. First, we soaked the seeds in water for 24 h. Then, we carefully cut away a small piece of each seed before submerging the seeds in the tetrazolium solution at 25 °C for 48 h. Seeds stained red or pink were considered to be viable, while unstained seeds were considered to be non-viable.

For each inundation event, we estimated the percent germination per fruit, which was defined as the number of germinated seeds/total number of seeds per fruit. In subsequent inundation events, we also estimated the cumulative percent germination per fruit, which was the sum of the germinated seeds from the first trial to the current trial/total number of seeds per fruit. At the end of the experiment, we estimated the total percent germination by calculating the mean percent germination across all the fruits for each morphotype.

For *Callitriche brutia*, a single inundation event was performed, using a procedure similar to the one described above for the G1 event with *R. peltatus* subsp. *sanicunifolius*. Furthermore, as above, the seeds representing terrestrial morphotype plants came from individuals that had grown naturally in dry temporary ponds. In contrast, the seeds representing aquatic morphotype plants came from individuals grown in tanks using sediments from natural temporary ponds. After a 6-to-8-month inundation period, we harvested the plants and collected their seeds. All the seeds were stored under dry conditions at room temperature (20–26 °C) until the inundation event. We obtained 249 seeds from aquatic morphotype plants and 341 seeds from terrestrial morphotype plants. We mixed all the seeds in each group together to estimate the total percent germination, which was defined as the total number of germinated seeds/the total number of seeds used in the experiment for the aquatic versus the terrestrial morphotype plants. After the inundation event, we examined the remaining ungerminated seeds and classified them on the basis of their appearance. There were two groups: seeds that appeared to be undamaged and seeds that appeared to be damaged (e.g., infected with fungi or partially germinated).

### 2.4. Simulated Seed Production under Different Pond Inundation Conditions

We estimated the number of seeds that could theoretically be produced in wet and dry years by using the estimates of percent germination we obtained for the aquatic and terrestrial morphotypes of *R. peltatus* subsp. *saniculifolius*. We explored five different scenarios: (a) a series of four wet years during which the ponds were flooded (wwww) and only the aquatic morphotype was produced; (b) an initial dry year during which there were only terrestrial morphotype plants producing seeds, followed by three wet years during which aquatic morphotype plants produced seeds (dwww); (c) two dry years followed by two wet years (ddww); (d) three dry years followed by one wet year (dddw); and (e) four dry years (dddd). We began with a set of 100 seeds coming from aquatic morphotype individuals with 100% survival. Because the wet area of the ponds, and thus the area in which seeds can germinate, may decrease in dry years, we utilized three different estimates of the number of terrestrial morphotype plants relative to the number of aquatic morphotype plants occurring in wet years (100%: the wet area is the same as in wet years, and the number of terrestrial morphotype plants is equal to the number of aquatic morphotype plants that grow in a wet year; 50%: the wet area is reduced to one-half of the pond basin, and the number of terrestrial morphotype plants is one-half of the number of aquatic morphotype plants that grow in a wet year; and 10%: the wet area is reduced to **^1^/_10_** of the pond basin, and the number of terrestrial morphotype plants is **^1^/_10_** of the number of aquatic morphotype plants that grow in a wet year). The number of seeds produced every year was estimated by multiplying the number of plants by percent germination, where annual mean fruit production for each plant was 4, and each fruit contained 20 seeds (see Table S1 for more details).

### 2.5. Statistical Analyses

For *R. peltatus* subsp. *saniculifolius*, we obtained the number of germinated and ungerminated seeds for each fruit during each inundation event. Then, we calculated the total percent germination using the mean percent germination for each fruit during each inundation event. For *C. brutia*, we had data for the individual seeds: binomial data indicating whether the seed germinated (1) or not (0) and categorical data indicating whether the seed came from a plant with the aquatic or terrestrial morphotype. To detect differences in germination between the two morphotypes, we used generalized linear models (GLM) with quasibinomial error distribution (i.e., due to overdispersion) in which the response variable was the binomial germination status (germinated vs. ungerminated). For *R. peltatus* subsp. *saniculifolius*, the total number of germinated versus ungerminated seeds per fruit was the response variable; plant morphotype (aquatic vs. terrestrial) and inundation event (G1–G5) were the explanatory variables. Tukey tests were then used to perform post-hoc paired comparisons. Similarly, we carried out a GLM (with a binomial error distribution) in which the response variable was the number of viable versus non-viable seeds for *R. peltatus* subsp. *saniculifolius* and the explanatory variable was plant morphotype. For *C. brutia*, we carried out a GLM (with a quasibinomial error distribution) in which the appearance of the ungerminated seeds was the response variable (undamaged vs. damaged) and plant morphotype was the explanatory variable. To analyze differences in time to germination, we carried out separate GLMs for the inundation events G1, G2, and G3. In the G2 models, we used a Poisson error distribution; in the G1 and G3 models, we used a quasi-Poisson error distribution (i.e., due to overdispersion). All the statistical analyses were performed using R [37].

## 3. Results

### 3.1. Germination Patterns of Ranunculus peltatus subsp. saniculifolius

**Figure 3:**
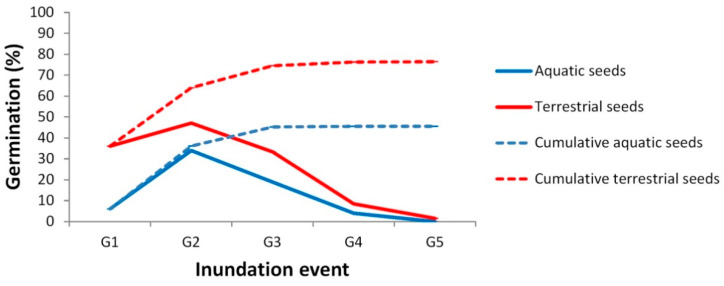
Event-specific and cumulative percent germination for *Ranunculus peltatus* subsp. *saniculifolius* seeds produced by aquatic and terrestrial morphotypes.

In *Ranunculus peltatus* subsp. *saniculifolius*, seed germination differed significantly between morphotypes (F_1, 279_ = 9.838, *p* = 0.0019) and among inundation events (F_1, 279_ = 54.24, *p* < 0.0005); also, the interaction of the two variables was significant (F_4, 279_ = 8.38, *p* < 0.0005). During the first inundation event, percent germination was 6-fold higher for seeds from terrestrial morphotype plants (hereafter, terrestrial seeds) (mean = 36.1%, standard error = +6.0%) than from seeds from aquatic morphotype plants (hereafter, aquatic seeds) (mean = 6.1%, standard error = +3.1%) (Tukey test, *p* = 0.022). Percent germination for the aquatic seeds was higher during the second inundation event than during the first inundation event (Tukey test: *p* = 0.0004), and percent germination peaked for both the aquatic and terrestrial seeds during the second inundation event (Figure 3). This maximum percent germination for the aquatic seeds did not differ statistically from the percent germination for the terrestrial seeds in the first inundation event (Tukey test, *p* = 0.999). Across all the inundation events, the terrestrial seeds had higher event-specific and cumulative percent germination than did the aquatic seeds (Figure 3).

Considering the total number of seeds in the experiment, cumulative percent germination differed significantly between morphotypes (F_1, 280_ = 46.66, *p* < 0.0005) and among inundation events (F_4, 280_ = 14.55, *p* < 0.0005), but the interaction between the variables was not significant. Pairwise comparison revealed that the percent germination for the aquatic and terrestrial seeds differed exclusively during G1 (Tukey test, *p* = 0.017), and the difference between the cumulative percent germination of both groups persisted in the subsequent inundation events (Figure 3). The percent germination for the aquatic seeds in G1 differed from the cumulative percent germination for the aquatic seeds during all the other inundation events (*p* < 0.05 for the four paired comparisons). The same was true for the terrestrial seeds (*p* < 0.005), except that the difference in the percent germination between G1 and G2 was marginally insignificant (*p* = 0.07).

The total percent germination was 29.7% higher, on average, for the terrestrial seeds than for the aquatic seeds (F_1, 56_ = 8.76, *p* = 0.0045). 

After the five inundation events, all the seeds had germinated for 24.1% of fruits from the terrestrial morphotype plants and for 17.2% of fruits from the aquatic morphotype plants. The percentage of ungerminated seeds that remained viable was low for both the aquatic morphotype (mean = 5.0%, standard error = + 2.3%) and the terrestrial morphotype (mean = 0.6%, standard error = + 0.4%), and the difference between the groups was not significant (χ^2^ = 1333.0, *p* = 0.1441).

The seeds germinated 5–40 days after inundation (Table 2). During the first and third inundation events, the aquatic and terrestrial seeds did not differ in their date of germination. However, during the second inundation event, when all the seeds germinated faster and within a shorter time period, the aquatic seeds germinated significantly earlier than the terrestrial seeds (F_1, 171_ = 6.71, *p* = 0.010, Table 2).

### 3.2. Germination Patterns in Callitriche brutia

**Figure 4:**
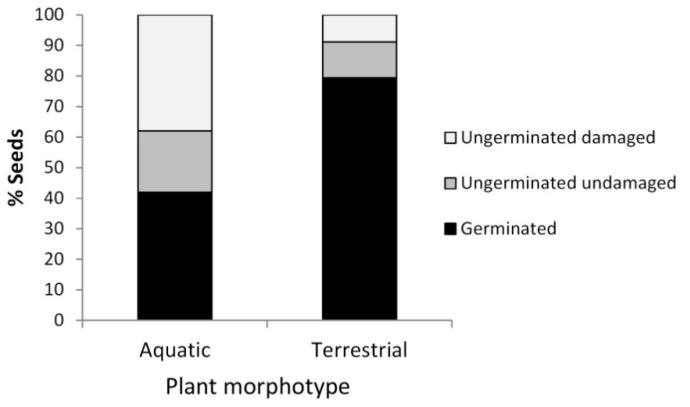
Percentage of germinated and ungerminated *Callitriche brutia* seeds at the end of the inundation experiment (aquatic morphotype seeds = 587, terrestrial morphotype seeds = 214). The ungerminated seeds were visually assessed and classified as undamaged or damaged.

Percent germination was significantly higher for the terrestrial seeds (79.5%) than for the aquatic seeds (41.9%) (F_1, 800_ = 91.99, *p* < 0.0005, Figure 4). However, time to germination did not differ between the groups: the terrestrial seeds germinated 7.3 + 0.21 days after inundation, while the aquatic seeds germinated 8.9 + 0.41 days after inundation.

After the inundation event, when only ungerminated seeds remained, the percentage of terrestrial seeds that appeared to be undamaged (11.7%) was significantly different from the percentage of aquatic seeds that appeared to be undamaged (20.1%) (F_1, 380_ = 7.93, *p* = 0.0051).

### 3.3. Simulated Seed Production under Different Pond Inundation Conditions 

**Figure 5:**
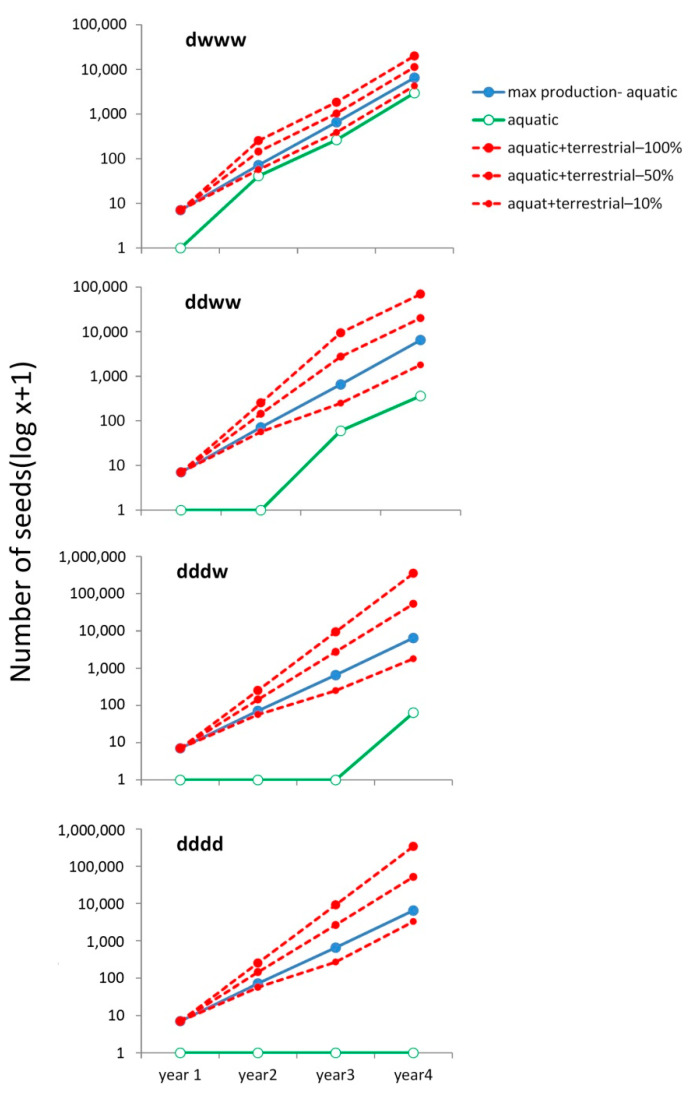
Results of the *Ranunculus peltatus* subsp. *saniculifolius* seed production simulations. The starting number of seeds was 100. Seed production patterns were then modeled across four years with different sets of climatic conditions (dwww: one dry year followed by three wet years; ddww: two dry years followed by two wet years; dddw: three dry years followed by a wet year; dddd: four dry years). Annual seed production was estimated using the percent germination values from the experiment. Percent germination values for aquatic morphotype plants were applied in wet years (G1 = 0.0606, G2 = 0.3397, G3 = 0.1892, and G4 = 0.04), and percent germination values for terrestrial morphotype plants were applied in dry years (G1 = 0.3605, G2 = 0.4711, G3 = 0.3332, and G4 = 0.0850). It was assumed that the survival of the aquatic morphotype plants was 100%. The simulations also examined the effect of reducing the area available for terrestrial morphotype growth by setting the relative size of the terrestrial morphotype population (i.e., the number of terrestrial morphotype plants relative to the number of aquatic morphotype plants that grow in a wet year) to 100%, 50%, or 10%. For the sake of comparison, maximum seed production by aquatic morphotype plants across four wet years (blue line) has been included in each figure.

The results of the models of seed production by *Ranunculus peltatus* subsp. *saniculifolius* are shown in Figure 5. When only the aquatic morphotype reproduced, there was no seed production in dry years, and thus, no contribution to the seed bank. Under real-life conditions, the seed bank would deteriorate or become depleted over time if seedlings emerged but did not successfully reproduce. When seeds were produced by the terrestrial morphotype, the total seed production by the terrestrial and aquatic morphotypes after consecutive dry years was always larger than the maximum seed production by the aquatic morphotypes alone after consecutive wet years, except for when the relative size of the terrestrial morphotype population was 10%. However, even in such scenarios, seed production remained high and resembled the maximum seed production by the aquatic morphotype alone (see Table S1 for details on how the seed numbers in Figure 5 were calculated).

## 4. Discussion

In this study, we explored the mechanisms used by submerged aquatic plants to persist in unpredictable temporary habitats. One strategy is to produce seeds with different dormancy periods that germinate during different inundation periods. A second strategy is to develop terrestrial morphotypes that can produce seeds during dry periods; these seeds then display a high level of germination during the subsequent wet period, allowing aquatic species to re-establish their populations, even if the seed bank has been depleted.

### 4.1. Seed Banks

Because temporary ponds usually experience annual dry periods, seed banks help plant populations become re-established when conditions become wet again. In our study of *R. peltatus* subsp. *saniculifolius*, the percent germination was low during the first inundation event that followed seed production. This species has been described as “annual or perhaps perennial” [27], since the vegetative parts of the plant may persist over several annual cycles. The low percent germination seen during the first inundation event could have been influenced by the plants’ ability to use vegetative reproduction to persist during years without the usual summer dry phase. We found that germination peaked during the second inundation event, which means that the seed bank could help restore local populations after dry periods and still retain a large number of ungerminated viable seeds for the future. Cross et al. [13] also observed differences in the germination patterns of different aquatic plant species across three consecutive annual cycles, which resulted in sediments containing seeds of different ages and in different stages of dormancy—a seed bank allowing for plant community resilience in the face of unpredictable reproductive conditions. Our findings for *R. peltatus* subsp. *saniculifolius* suggest that seeds produced in the same year have different dormancy characteristics and require different stimuli for seedling emergence. Given that germination peaked after two years, it seems likely that the plants could persist even in the event of subsequently unfavorable conditions, although a small percentage of seeds might germinate in the years before and after. Such a bet-hedging strategy could help guarantee reproduction in the intermediate and long term, via the production of a certain percentage of seeds displaying longer dormancy and the creation of a persistent seed bank composed of seeds of different ages.

Bet-hedging strategies in annual desert plants reduce the risk of complete reproductive failure in years characterized by adverse conditions [38,39]. Similarly, aquatic plants are able to cope with the unpredictability of Mediterranean temporary wetlands by spreading out annual seed production over numerous years. The marked persistence of seed banks thus arises not only from seed accumulation across different annual cycles, but also from the different germination patterns of seeds produced within a given year, which help diversify germination dynamics over time. Thus, this strategy also increases the likelihood that a population will persist starting from its arrival in a new habitat, prior to seed bank formation.

### 4.2. Terrestrial Plant Morphotypes

Terrestrial morphotypes of submerged aquatic plants can produce fruits and seeds, helping to restore populations after dry periods. In addition to helping build the seed bank, the ability to generate a terrestrial morphotype capable of reproduction also serves to reduce the risk of species extinction. As a consequence, aquatic plants can produce seeds even under dry conditions. Our results also show that percent germination was higher for seeds produced by terrestrial morphotype plants than for seeds produced by the aquatic morphotype plants. This higher percent germination contrasted with the more typical germination dynamics seen in aquatic plants and likely helps accelerate population re-establishment, compensating for reproductive failure in dry years. Percent germination for seeds from both terrestrial and aquatic plants peaked two years after seed production. However, the main difference between the two groups of seeds was that those from the terrestrial morphotype also displayed a high level of germination during the first year; such dynamics could help rapidly restore populations after a bad year. In our simulations of *R. peltatus* subsp. *saniculifolius* seed production, we examined population-level trends by comparing seed production for the aquatic morphotype alone versus for the aquatic and terrestrial morphotypes together. Aquatic plants would eventually die off if no seed production occurred in dry years. If reproduction failed over several years, the persistence of the population would rely solely on a very depleted (and perhaps exhausted) seed bank, which would contain old, mainly deteriorated seeds. It is probable that only a small percentage would remain viable, given that, in our experiment, 2.3% of the ungerminated *R. peltatus* subsp. *saniculifolius* seeds remaining after the five inundation events were still viable. Thanks to the seeds produced by terrestrial morphotype plants, reproductive failures experienced by aquatic morphotype plants because of unfavorable conditions can be compensated for the following year. In our simulations, we even observed an increase in seed production that was maintained across subsequent years. Total seed production in consecutive dry years should be largely dependent on the number of mature terrestrial morphotype plants. Such plants may be constrained to live in the areas of dry ponds that have suitable levels of moisture, which may vary considerably among ponds. However, even in the worst-case scenario that we simulated, where the relative size of the terrestrial morphotype population was 10%, the complementary production of seeds by the latter nonetheless boosted seed production, and thus, population stability, even during dry years.

Terrestrial morphotypes are common in submerged aquatic plant species that face fluctuating water levels. This plasticity is considered to be an adaptation that allows species to cope with dry periods; indeed, *Myriophyllum variifolium* populations can even deal with extreme environmental conditions [19]. Being able to switch between aquatic and terrestrial morphotypes does more than simply allow plants to persist in the face of pond drying. It also has long-term advantages because it replenishes the seed bank, which would otherwise become depleted. In particular, *R. peltatus* subsp. *saniculifolius* has adapted to cope with dry conditions and can swiftly change morphotype depending on local hydrological conditions [17,35]. Although the terrestrial morphotype is smaller, its level of fruit production is similar to that of the aquatic morphotype because its reproductive period is longer [17].

For most of the plant species found in Doñana temporary ponds, seed germination is primarily triggered by inundation and is influenced by the number of days since inundation [40]. This trait allows plants to synchronize their annual cycle with the pond’s inundation phase, which is unpredictable. Here, we found that *R. peltatus* subsp. *saniculifolius* germination occurred earlier during the second inundation event, a period in which a greater investment in germination was observed. Plants with earlier-germinating seeds may even increase their reproductive success because they prolong the growth period, which serves to increase seed production and the length of the reproductive period.

In the case of *C. brutia*, we analyzed seed germination during a single inundation event following a dry phase. The main difference between *R. peltatus* subsp. *saniculifolius* and *C. brutia* was that the latter species displayed a high level of germination even during that single indundation event. *Callitriche brutia* is an annual herbaceous plant [29] and likely depends exclusively on seed germination to re-establish its populations. Therefore, seedling production must be high during every annual cycle. On the other hand, like *R. peltatus* subsp. *saniculifolius*, *C. brutia* also displayed early seed germination and adopted a terrestrial morphotype to ensure seed production during dry years. In addition, percent germination was higher for terrestrial seeds, which could compensate for population declines during dry years. With such high levels of germination, *C. brutia* would seem to store a smaller percentage of annual seed production in the seed bank than would *R. peltatus* subsp. *saniculifolius*. This percentage may be quite small during dry years, which indicates that, in *C. brutia*, seed production by terrestrial forms is essential for the long-term persistence of populations. 

On the basis of our results, it appears that the capacity to persist through dry periods and the ability to develop terrestrial morphotypes are strategies used by submerged plant species to weather the unpredictable conditions in temporary wetlands. In Doñana temporary ponds, terrestrial morphotypes are seen in most of the common submerged plant species, such as *R. peltatus*, *C. brutia*, *C. stagnalis*, *C. lusitanica*, *C. obtusangula*, *Potamogeton polygonifolius*, and *Myriophyllum alterniflorum*. In all these species, terrestrial morphotypes likely serve to replenish the seed bank during dry periods, and probably also boost germination levels when wet conditions return, promoting the re-establishment of aquatic plant populations.

## Figures and Tables

**Table 1:** Description of the conditions under which *Ranunculus peltatus* subsp. *saniculifolius* and *Callitriche brutia* seeds were stored before the inundation events. The dates for each inundation event are also indicated.

Species	Inundation Event	Seed Storage Conditions	Start Date	End Date
*Ranunculus peltatus* subsp. *saniculifolius*	G1	Dry, room temperature (20–26 °C)	04/12/2012	21/01/2013
	G2	Dry, 4 °C	16/05/2013	25/06/2013
	G3	Dry, room temperature (20–26 °C)	08/01/2014	19/02/2014
	G4	Dry, 4 °C	11/11/2014	12/12/2014
	G5	Dry, 10 °C	08/05/2015	22/06/2015
*Callitriche brutia*	G1	Dry, room temperature (20–26 °C)	23/01/2013	13/03/2013

**Table 2:** Number of days from the start of the inundation event to the germination of *Ranunculus peltatus* subsp. *saniculifolius* seeds produced by aquatic and terrestrial morphotypes across the five inundation events. The mean, standard error (SE), minimum, and maximum are indicated. Seed number (N) is also given.

Inundation Event	Plant Morphotype	Mean	SE	Min	Max	N
G1	Aquatic	14.18	1.71	8	40	22
	Terrestrial	12.44	0.50	8	37	156
G2	Aquatic	6.45	0.28	5	18	82
	Terrestrial	7.90	0.48	5	25	91
G3	Aquatic	10.69	0.87	9	19	13
	Terrestrial	10.21	0.69	9	23	28
G4	Aquatic	14.0	-	-	-	1
	Terrestrial	14.25	2.56	7	31	8
G5	Aquatic	-	-	-	-	0
	Terrestrial	14.0		1	1	1

## Data Availability

Not Applicable.

## Supplementary Materials

The following are available online at https://www.mdpi.com/article/10.3390/plants10040741/s1, Table S1: Simulation of *Ranunculus peltatus* subsp. *saniculifolius* seed production over four consecutive years with different sets of climatic conditions.

## References

[1] Barrett S.C.H., Eckert C.G., Husband B.C. (1993). Evolutionary processes in aquatic plant populations. Aquat. Bot..

[2] Schulthorpe C.D. (1967). The Biology of Aquatic Vascular Plants.

[3] Cronk J.K. (2001). , Fennessy, M.S. Wetland Plants: Biology and Ecology.

[4] Díaz-Paniagua C., Fernández-Zamudio R., Florencio M., García-Murillo P., Gómez-Rodríguez C., Portheault A., Serrano L., Siljeström P. (2010). Temporary ponds from Doñana National Park: A system of natural habitats for the preservation of aquatic flora and fauna. Limnetica.

[5] Díaz-Paniagua C., Fernandez-Zamudio R., Serrano L., Florencio M., Gómez-Rodríguez C., Sousa A., Sánchez Castillo P., García-Murillo P., Siljeström P. (2015). El Sistema de Lagunas Temporales de Doñana, una Red de Hábitats Acuáticos Singulares.

[6] Bonis A., Lepart J., Grillas P. (1995). Seed bank dynamics and coexistence of annual macrophytes in a temporary and variable habitat. Oikos.

[7] Brock M.A., Nielsen D.L., Shiel S.J., Green J.D., Langley J.D. (2003). Drought and aquatic community resilience: The role of eggs and seeds in sediments of temporary wetlands. Freshw. Biol..

[8] Warwick N.W.M., Brock M.A. (2003). Plant reproduction in temporary wetlands: The effects of seasonal timing, depth, and duration of flooding. Aquat. Bot..

[9] Deil U. (2005). A review on habitats, plant traits and vegetation of ephemeral wetlands—A global perspective. Phytocoenologia.

[10] Aponte C., Kazakis G., Ghosn D., Papanastasis V.P. (2010). Characteristics of the soil seed bank in Mediterranean temporary ponds and its role in ecosystem dynamics. Wetl. Ecol. Manag..

[11] Brock M.A. (2011). Persistence of seed banks in Australian temporary wetlands. Freshw. Biol..

[12] Carta A. (2016). Seed regeneration in Mediterranean temporary ponds: Germination ecophysiology and vegetation processes. Hydrobiologia.

[13] Cross A.T., Turner S.R., Renton M., Baskin J.M., Dixon K.W., Merritt D.J. (2015). Seed dormancy and persistent sediment seed banks of ephemeral freshwater rock pools in the Australian monsoon tropics. Ann. Bot..

[14] Grillas P., Garcia-Murillo P., Geertz-Hansen O., Marbá N., Montes C., Duarte C.M., Tan Ham L., Grossmann A. (1993). Submerged macrophyte seed bank in a Mediterranean temporary marsh: Abundance and relationship with established vegetation. Oecologia.

[15] Rhazi L., Grillas P., Tan Ham L., El Khyari D. (2001). The seed bank and the between years dynamics of the vegetation of a Mediterranean temporary pool (NW Morocco). Ecol. Mediterr..

[16] Thompson K., Fenner M. (1992). The functional ecology of seed banks. Seeds, the Ecology of Regeneration in Plant Communities.

[17] Volder A., Bonis A., Grillas P. (1997). Effects of drought and flooding on the reproduction of an amphibious plant, *Ranunculus peltatus*. Aquat. Bot..

[18] Brock M.A., Casanova M.T., Klomp N., Lunt I. (1997). Plant life at the edges of wetlands: Ecological responses to wetting and drying patterns. Frontiers in Ecology: Building the Links.

[19] Brock M.A. (1991). Mechanisms for maintaining persistent populations of *Myriophyllum variifolium* in a fluctuating Australian shallow lake. Aquat. Bot..

[20] Cox J. (1997). Terrestrial form of Myriophyllum alterniflorum. Bot. Soc. Brit. Isles News.

[21] Ritter N.P., Crow G.E. (1998). *Myriophyllum quitense* Kunth (Haloragaceae) in Bolivia: A terrestrial growth-form with bisexual flowers. Aquat. Bot..

[22] Lansdown R.V. (1999). A terrestrial form of *Callitriche truncata* Guss. subsp. occidentalis (ROUY) BRAUN-BLANQUET (Callitrichaceae). Watsonia.

[23] Leck M.A., Brock M.A. (2000). Ecological and evolutionary trends in wetlands: Evidence from seeds and seed banks in New South Wales, Australia and New Jersey, USA. Plant Species Biol..

[24] Kaplan Z. (2002). Phenotypic plasticity in *Potamogeton* (Potamogetonaceae). Folia Geobot..

[25] Fernández-Zamudio R., García-Murillo P., Díaz-Paniagua C. (2016). Physical and chemical features and water permanence determine aquatic plant distribution in a temporary pond network (Doñana National Park). Hydrobiologia.

[26] García-Murillo P., Fernández-Zamudio R., Díaz-Paniagua C., Coord (2015). Las plantas de las lagunas temporales de Doñana. El Sistema de Lagunas Temporales de Doñana, una Red de Hábitats Acuáticos Singulares.

[27] Cook C.D.K. (1966). A monographic study of *Ranunculus* subgenus *Batrachium* (DC.) A.Gray. Mitt. Bot. Staatssummlung Mȕnchen.

[28] Preston C.D., Croft J.M. (1997). Aquatic Plants in Britain and Ireland.

[29] Lansdown R.V. (2008). Water-starworts (Callitriche) of Europe.

[30] García-Murillo P., Castroviejo S., Morales R., Quintanar A., Cabezas F., Pujadas A.J., Cirujano S. (2010). Callitriche. Flora Iberica.

[31] Wiegleb G., Bobrov A.A., Zalewska-Gałosz J. (2017). A taxonomic account of *Ranunculus* section *Batrachium* (Ranunculaceae). Phytotaxa.

[32] Cirujano S., Meco A., García-Murillo P. (2014). Flora Acuática Española. Hidrófitos Vasculares.

[33] Baskin C.C., Baskin J.M. (1998). Seeds: Ecology, Biogeography, and Evolution of Dormancy and Germination.

[34] Velayos M. (1988). Acotaciones a *Ranunculus* subgénero *Batrachium* (DC) A. Gray: Tratamiento taxonómico general y estudio de la variabilidad de *R. peltatus*. An. Jard. Bot. Madr..

[35] Garbey C., Thiébaut G., Muller S. (2004). Morphological plasticity of a spreading aquatic macrophyte, *Ranunculus peltatus*, in response to environmental variables. Plant Ecol..

[36] Porter R., Durrell M., Romm H. (1947). The use of 2,3,5-triphenyl-tetrazolium chlorid as a measure of seed germinability. Plant Physiol..

[37] R Core Development Team (2019). R: A Language and Environment for Statistical Computing.

[38] Venable D.L., Lawlor L. (1980). Delayed germination and dispersal in desert annuals—Escape in space and time. Oecologia.

[39] Gremer J.R., Venable D.L. (2014). Bet hedging in desert winter annual plants: Optimal germination strategies in a variable environment. Ecol. Let..

[40] Fernández-Zamudio R., García-Murillo P., Díaz-Paniagua C. (2018). Effect of the filling season on aquatic plants in Mediterraneantemporary ponds. J. Plant Ecol..

