# Supplementary material for: Terrestrial Morphotypes of Aquatic Plants Display Improved Seed Germination to Deal with Dry or Low-Rainfall Periods
Source: Plants (Basel). 2021 Apr 10;10(4):741. doi: 10.3390/plants10040741 (PMC8069061; doi:10.3390/plants10040741)
Supplement: Supplementary file 1 [file plants-10-00741-s001.pdf]

plants

Supplementary material

Table S1

We simulated *Ranunculus peltatus* subsp. *saniculifolius* seed production over four consecutive years with different sets of climatic conditions. We began with 100 seeds.

In the simultations, in wet years, all the plants had the aquatic morphotype and contributed to seed production across the four years (respective percent germination: GW1, GW2, GW3, and GW4).

In dry years, the plants had the terrestrial morphotype and contributed to seed production across the four years (respective percent germination: GT1, GT2, GT3, and GT4).

To calculate the total number of seeds produced every year (NS1–NS5), we first estimated the number of plants that appeared each year (e.g., A0, A0A1+B0, A0B1+A0A1A1+B0A1+C0) based on the product of the following variables: the number of seeds produced the previous year and the percent germination of those seeds (GW1–GW4 or GT1–GT4), plant survival, the number of seeds produced per plant (NS), and the relative size of the terrestrial morphotype population (PR) for the current year. This latter variable aimed to simulate the effect of reducing the area available for terrestrial morphotype growth. Plant survival (S) was 100% in both wet and dry years, and thus the number of seeds produced per year was equal to the number of plants in the following year. Each plant produced 4 fruits containing 25 seeds each (NS = 100). In wet years, there was no reduction in PR. In dry years, there were three different scenarios for PR: 100%, 50%, and 10%. These calculations are summarized in the table below:

Year 1	Year 2	Year 3	Year 4	Year 5
A0=100*G1	A0A1=A0*G1*S*NS*PR	A0A1A1=B0A1*G1*S*NS*PR	A0A1A1A1=A0A1A1*G1*S*NS*PR	A0A1A1A1A1=A0A1A1A1*G1
				A0A1A1B1=A0A1A1*G2*S*NS*PR
			A0A1B1=A0A1*G2*S*NS*PR	A0A1B1A1=A0A1B1*G1*S*NS*PR
				A0A1C1=A0A1*G3*S*NS*PR
		A0B1=A0*G2*S*NS*PR	A0B1A1=A0B1*G1*S*NS*PR	A0B1A1A1=A0B1A1*G1*S*NS*PR
				A0B1B1=A0B1*G2*S*NS*PR
			A0C1=A0*G3*S*NS*PR	A0C1A1=A0C1*G1*S*NS*PR
				A0D1=A0*G4*S*NS*PR
	B0=100*G2	B0A1=B0*G1*S*NS*PR	B0A1A1=B0A1*G1*S*NS*PR	B0A1A1A1=B0A1A1*G1*S*NS*PR
				B0A1B1=B0A1*G2*S*NS*PR
			B0B1=B0*G2*S*NS*PR	B0B1A1=B0B1*G1*S*NS*PR
				B0C1=B0*G3*S*NS*PR
		C0=100*G3	C0A1=C0*G1*S*NS*PR	C0A1A1=C0A1*G1
				C0B1=C0*G2*S*NS*PR
			D0=100*G4	D0A1=D0*G1*S*NS*PR

G: percent germination (aquatic: GW1–GW4, terrestrial: GT1–GT4); S: plant survival; NS: number of seeds produced per plant; PR: relative size of the terrestrial morphotype population.

In the simulations, the starting set of seeds came from aquatic morphotype plants, and we calculated the number of seeds in the first generation using percent germination for the aquatic seeds (GW1–GW4). For the next generations, we used the percent germination for the terrestrial morphotype plants if the year was dry (GT1–GT3) and the aquatic morphotype plants if the year was wet (GW1–GW4).

The number of plants produced by the initial set of seeds that germinated in the first year was A0, which was estimated using the product of the number of initial seeds and first-year percent germination (GW1, since the initial seeds came from aquatic morphotype plants). In the second year, plants came from the initial seeds—B0—and plant

number was calculated as for A0, but using second-year percent germination (GW2). There were also plants that came from A0—A0A1 plants—whose number was based on the product of A0; first-year percent germination (G1), which was different for the aquatic morphotype (GW1, if the plants emerged during a wet year) and for the terrestrial morphotype (GT1, if the plants emerged during a dry year); plant survival (100%); the number of seeds per plant; and the relative size of the terrestrial morphotype population. In the third year, plants came from the initial seeds—C0—calculated using GW3; from A0A1—the A0A1A1 plants—calculated using GW2 or GT2; and from B0—the A0B1 plants—calculated using GW1, and so on (see the details in the table).

In dry years, we used percent germination for the seeds produced by the terrestrial morphotype (i.e., GT1–GT4 instead of GW1–GW4). If the first year was dry, in the next round of seed production, we used the percent germination GW1–GW3, since the new plants produced by the terrestrial morphotype seeds would grow into aquatic morphotype plants. Similarly, shifts from GW to GT occurred in all the subsequent dry years. In the scenario with the four dry years, percent germination for the aquatic morphotype seeds was only used in the case of the initial seed set. For the four years of the simulation, percent germination for the terrestrial morphotype seeds was used. The seeds produced by B0, C0, and D0 were calculated using GW1, as with the initial seed set.

Additionally, for the sake of comparison, we calculated the number of seeds that would have been produced exclusively by aquatic plants in a scenario with four wet years.
